# Selective Enrichment of Munc13-2 in Presynaptic Active Zones of Hippocampal Pyramidal Cells That Innervate mGluR1α Expressing Interneurons

**DOI:** 10.3389/fnsyn.2021.773209

**Published:** 2022-02-10

**Authors:** Noemi Holderith, Mohammad Aldahabi, Zoltan Nusser

**Affiliations:** ^1^Institute of Experimental Medicine, Eotvos Lorand Research Network, Budapest, Hungary; ^2^János Szentágothai School of Neurosciences, Semmelweis University, Budapest, Hungary

**Keywords:** hippocampus, CA1, O-LM interneuron, paired recordings, multiplexed postembedding immunohistochemistry, vesicle release probability, short-term plasticity

## Abstract

Selective distribution of proteins in presynaptic active zones (AZs) is a prerequisite for generating postsynaptic target cell type-specific differences in presynaptic vesicle release probability (P_v_) and short-term plasticity, a characteristic feature of cortical pyramidal cells (PCs). In the hippocampus of rodents, somatostatin and mGluR1α expressing interneurons (mGluR1α+ INs) receive small, facilitating excitatory postsynaptic currents (EPSCs) from PCs and express Elfn1 that trans-synaptically recruits mGluR7 into the presynaptic AZ of PC axons. Here we show that Elfn1 also has a role in the selective recruitment of Munc13-2, a synaptic vesicle priming and docking protein, to PC AZs that innervate mGluR1α+ INs. In Elfn1 knock-out mice, unitary EPSCs (uEPSCs) in mGluR1α+ INs have threefold larger amplitudes with less pronounced short-term facilitation, which might be the consequence of the loss of either mGluR7 or Munc13-2 or both. Conditional genetic deletion of Munc13-2 from CA1 PCs results in the loss of Munc13-2, but not mGluR7 from the AZs, and has no effect on the amplitude of uEPSCs and leaves the characteristic short-term facilitation intact at PC to mGluR1α+ IN connection. Our results demonstrate that Munc13-1 alone is capable of imposing low P_v_ at PC to mGluR1α+ IN synapses and Munc13-2 has yet an unknown role in this synapse.

## Introduction

Postsynaptic target cell type-dependent differences in synaptic efficacy and short-term plasticity of excitatory synapses (Ali and Thomson, 1997, 1998; Reyes et al., 1998; Scanziani et al., 1998; Sun et al., 2005) have profound impacts on cortical network dynamics (Pouille and Scanziani, 2004). The first identified molecule with postsynaptic target cell type-dependent location in the presynaptic active zone (AZ) of pyramidal cells (PCs) was mGluR7. It was found to be selectively enriched in hippocampal PC AZs that innervate somatostatin (Som) and mGluR1α expressing interneurons (mGluR1α+ IN; Shigemoto et al., 1996) and its constitutive activity contributes to the low postsynaptic response amplitude of this synapse (Losonczy et al., 2003). Interestingly, mGluR7 is recruited into the AZ by Elfn1 (extracellular leucine-rich repeat and fibronectin type III domain containing 1) which is selectively expressed by Som/mGluR1α+ INs and located in the excitatory postsynaptic densities where Elfn1 trans-synaptically binds and activates mGluR7 (Sylwestrak and Ghosh, 2012; Tomioka et al., 2014; Stachniak et al., 2019). Ectopic expression of Elfn1 in parvalbumin expressing INs (PV+ INs) in the hippocampus changed the short-term plasticity from depression to moderate facilitation through an unknown mechanism (Sylwestrak and Ghosh, 2012).

Although presynaptic neurotransmitter receptors could powerfully influence neurotransmitter release and short-term plasticity, in the presence of a large number of presynaptic receptor blockers synapses still show very diverse functional properties. This diversity is likely the consequence of the heterogeneous molecular components of the AZ matrix that mediate synaptic vesicles (SVs) docking, priming, and release (Sudhof, 2012). Among these, members of the Munc13 protein family are essential for SV docking and priming (Augustin et al., 1999b; Varoqueaux et al., 2002; Siksou et al., 2007) and Munc13-containing supramolecular complexes constitute the docking/release sites in the AZs (Sakamoto et al., 2018). Three closely related Munc13 genes are present in mammals (Brose et al., 1995), out of which two, Munc13-1 and Munc13-2, are expressed in hippocampal PCs (Rosenmund et al., 2002). The C-terminal part of the proteins shares common domain structures, which is functionally essential for their priming activity and are structurally homologous to vesicle tethering factors (Basu et al., 2005; Stevens et al., 2005; Li et al., 2011). Munc13-1 has one while Munc13-2 has two major splice variants with different N-terminal regions: the brain-specific bMunc13-2 and the ubiquitously expressed ubMunc13-2 both of which are expressed in CA1 PCs (Brose et al., 1995; Augustin et al., 1999b; Betz et al., 2001; Breustedt et al., 2010; Kawabe et al., 2017). Experiments in cultured autaptic neurons suggest that Munc13-1 and Munc13-2 bestow different short-term plasticity to the synapses. In 90% of the axon terminals of cultured PCs, Munc13-1 primed vesicles have high P_v_ and the synapses display short-term depression while in 10% of boutons, the presence of Munc13-2, in the absence of Munc13-1, confers low P_v_ and short-term facilitation (Rosenmund et al., 2002). As this correlation appears to hold in other synapses (Cooper et al., 2012; Man et al., 2015), the following concept emerged: high P_v_ synapses that show short-term depression are equipped with Munc13-1 which enables tight docking of readily releasable SVs, while low P_v_ synapses that display short-term facilitation employ Munc13-2 and vesicles are loosely docked and require intracellular [Ca^2+^] increase to become release competent (Neher and Brose, 2018). Munc13-2 immunolabeling in the hippocampus showed an uneven distribution of the protein with strong staining in the stratum oriens of the CA1 area (Kawabe et al., 2017) where most of the dendrites of mGluR1α+ INs are located, raising the question whether the low P_v_ of CA1 PC to mGluR1α+ IN synapses could be the consequence of the presence of Munc13-2 as a priming factor?

## Materials and Methods

### Animals

Two young adult male Wistar rats (P30, 42), 3 adult (P50) male C57BL/6J mice, 47 adult (P50-60) Tg(Chrna2-Cre)OE25Gsat/Mmucd (RRID:MMRRC_036502-UCD, on C57BL/6J background (Leao et al., 2012) crossed with reporter line Ai9 [Gt(ROSA)26Sor_CAG/LSL_tdTomato], 15 adult (P50–70) male C57BL/6N-*Elfn1*^tm1.1(KOMP)Vlcg^**/MbpMmucd (RRID:MMRRC_047527-UCD, on C57BL/6N background (Tomioka et al., 2014) and 5 heterozygous littermate control mice, and 35 adult P50–70 C57BL/6N-*Unc13b^TM 1a(KOMP)Wtsi^*/MbpMmucd (RRID:MMRRC_050292-UCD, on C57BL/6N background) were used. Mice of both sexes were used. The animals were housed in the vivarium of the Institute of Experimental Medicine in a normal 12 h/12 h light/dark cycle and had access to water and food *ad libitum*. All the experiments were carried out in accordance with the Hungarian Act of Animal Care and Experimentation 40/2013 (II.14) and with the ethical guidelines of the Animal Committee of the Institute of Experimental Medicine, Budapest.

### Virus Injection

Mice were anesthetized with a mixture of ketamine, xylasine, pypolphene (0.625, 6.25, 1.25 mg/ml respectively, 10 μl/g body weight). The pAAV-Ef1a-mCherry-IRES-Cre was a gift from Karl Deisseroth (1.8 × 10^13^ Addgene viral prep # 55632-AAV8^1^ ; RRID:Addgene_55632) (Fenno et al., 2014) or pENN.AAV.CamKII 0.4.Cre.SV40 was a gift from James M. Wilson (Addgene viral prep # 105558-AAv9^2^ ; RRID:Addgene_105558) (1:10 dilution 2.8 × 10^13^, Penn Vector Core) were injected into the dorsal hippocampus (200 nl, coordinates from the Bregma in mm: antero posterior/dorso ventral/lateral: 2.1/1.1/1.3 and/or 2.2/1.5/1.2). After 2 weeks, the mice were either perfused or *in vitro* acute slices were prepared from the dorsal hippocampus as below.

### Slice Preparation and Electrophysiological Recordings

The following animals were used: 44 adult (P50-60) Tg(Chrna2-Cre)OE25Gsat/Mmucd (RRID:MMRRC_036502-UCD, on C57Bl/6J background) crossed with reporter line Ai9 [Gt(ROSA)26Sor_CAG/LSL_tdTomato]; 10 adult P50-70 C57BL/6N-*Elfn1*^tm1.1(KOMP)Vlcg^**/MbpMmucd. Nineteen adult P50-70 C57BL/6N-*Unc13b^TM 1a(KOMP)Wtsi^*/MbpMmucd mice were injected with pAAV-Ef1a-mCherry-IRES-Cre more than 2 weeks prior to the slice preparation. Mice were stably anesthetized with a ketamine, xylasine, and pypolphene cocktail (0.625, 6.25, 1.25 mg/ml, respectively, 10 μl/g body weight), and then decapitated; the brain was quickly removed and placed into an ice-cold cutting solution containing the following (in mM): sucrose, 205.2; KCl, 2.5; NaHCO_3_, 26; CaCl_2_, 0.5; MgCl_2_, 5; NaH_2_PO_4_, 1.25; and glucose, 10, saturated with 95% O_2_ and 5% CO_2_. Coronal slices (300 μm thick) were then cut from the dorsal hippocampus using a Leica Vibratome (VT1200S) and were incubated in a submerged-type holding chamber in an artificial cerebral spinal fluid (ACSF) containing the following (in mM): NaCl, 126; KCl, 2.5; NaHCO_3_, 26; CaCl_2_, 2; MgCl_2_, 2; NaH_2_PO_4_, 1.25; and glucose, 10, saturated with 95% O_2_ and 5% CO_2_, pH 7.2–7.4, at 36°C for 30 min, then kept at 22–24°C until use. Recordings were performed in the same ACSF supplemented with 2 μM AM251 to block presynaptic CB1 receptors at 32°C up to 6 h after slicing.

Cells were visualized using infrared differential interference contrast (DIC) imaging on a Nikon Eclipse FN1 microscope with a 40X water immersion objective (NA = 0.8). CA1 PCs were identified from their position and morphology, and the virally expressed mCherry was identified using fluorescent illumination. Whole-cell current-clamp recordings were performed from CA1 PCs using MultiClamp 700B amplifiers (Molecular Devices). Recorded traces were filtered at 3–4 kHz and digitized online at 50 kHz. Patch pipettes (resistance 3–6 MΩ) were pulled from thick-walled borosilicate glass capillaries with an inner filament. Intracellular solution contained the following (in mM): K-gluconate, 130; KCl, 5; MgCl_2_, 2; EGTA, 0.05; creatine phosphate, 10; HEPES, 10; ATP, 2; GTP, 1; and biocytin, 7; glutamate, 10 (for presynaptic PCs only) pH = 7.3; 290–300 mOsm. Pyramidal cells were held at –65 mV (with a maximum of ± 100 pA DC current) and trains of 3 APs at 40 Hz were evoked with 1.2–1.5 ms-long depolarizing current pulses (1.5–2 nA). Peak amplitude and full width at half-maximal amplitude of the APs were monitored and the cells were rejected if any of these parameters changed greater than 10%. Postsynaptic O-LM cells were identified in the stratum oriens of the CA1 region by tdTomato fluorescence in the Tg(Chrna2-Cre)OE25Gsat/Mmucd animals crossed with reporter line Ai9 or by their morphology and firing pattern obtained with de- or hyperpolarizing square current injections (600 ms, from –250 to 300 pA with 50 pA steps). Paired whole-cell recordings were performed with a dual-channel amplifier (MultiClamp 700B; Axon Instruments, CA, United States). Data were filtered at 3–4 kHz (Bessel filter), digitized online at 50 kHz, recorded and analyzed using pClamp 10.3 (Molecular Devices, CA, United States). Postsynaptic INs were held at –65 mV (with a maximum of ± 200 pA DC current) in the voltage-clamp mode with the access resistance below 20 MOhm.

### Tissue Processing

After recordings, the slices were fixed in a solution containing 4% formaldehyde (FA, Molar Chemicals, Budapest, Hungary), 0.2% picric acid in 0.1 M phosphate buffer (PB), pH = 7.4, at 4°C for 12 h. Immunolabeling was carried out without re-sectioning. Slices were washed in 0.1 M PB and blocked in normal goat serum (NGS, 10%; Vector Laboratories, CA, United States) for 1 h made up in Tris-buffered saline (TBS; pH 7.4), incubated in the solutions of the primary antibodies: guinea pig anti-mGluR1α (1:1,000, Frontier Institute Co., Ltd.; Cat# mGluR1a-GP-Af660, RRID:AB_2531897), or a cocktail of this Ab and a mouse anti-Cre Ab (IgG1, 1:1,000, Millipore, Cat# MAB3120, RRID:AB_2085748); diluted in TBS containing 2% NGS and 0.2% TritonX-100. Biocytin was visualized with Cy3-conjugated streptavidin (1:1,000; Jackson Immunoresearch Laboratories, PA, United States, RRID:AB_2337244). After several washes, the following secondary antibodies were applied: Alexa488-conjugated goat anti-mouse IgG1 (Jackson Immunoresearch Laboratories, PA, United States, Code: 115-547-185, RRID:AB_2632534), and Cy5-conjugated donkey anti-guinea pig IgGs (Jackson Immunoresearch, Code 706-175-148, RRID: AB_2340462). Sections were mounted in Vectashield. Image stacks were acquired with an Olympus FV1000 confocal microscope with 20x and 60x (oil immersion) objectives. A PC was considered virally infected if its nucleus had a detectable Cre signal. A cell was considered an O-LM cell, if the axon arborized in the stratum lacunosum-moleculare.

### Processing of Perfusion-Fixed Tissue

Two young-adult Wistar rats (P30, 42), 3 adult (P50) male C57BL/6J mice, 3 adult (P50–60) Tg(Chrna2-Cre)OE25Gsat/Mmucd, (RRID:MMRRC_036502-UCD, on C57BL/6J background) crossed with reporter line Ai9 [Gt(ROSA)26Sor_CAG/LSL_tdTomato], 5 adult (P50–70) male C57BL/6N-*Elfn1*^tm1.1(KOMP)Vlcg^**/MbpMmucd (RRID:MMRRC_047527-UCD, on C57BL/6N background) and 5 heterozygous littermate control mice, and 16 adult P50-70 C57BL/6N-*Unc13b^TM 1a(KOMP)Wtsi^*/MbpMmucd (RRID:MMRRC_050292-UCD, on C57BL/6N background) were deeply anesthetized and transcardially perfused with a fixative containing 1% FA (Molar Chemicals, Budapest, Hungary) and 0.2% picric acid in 0.1 M PB or in 0.1 M sodium acetate buffer for 15–20 min. The brains were then quickly removed from the skull and placed in 0.1 M PB. Next, 60–100 μm thick sections were cut from the dorsal hippocampus. After blocking in NGS or normal donkey serum (NDS, 10%) for 1 h made up in TBS, the sections were incubated in the following primary antibodies: guinea pig anti-mGluR1α (1:1,000; Frontier Institute Co., Ltd, Nittobo Medical Co. Ltd, Tokyo, Japan; Cat# mGluR1a-GP-Af660, RRID:AB_2531897), goat anti-mGluR1α (1:1,000, Frontier Institute Co., Ltd; Cat# mGluR1a-Go-Af1220, RRID:AB_2571800), rabbit anti-Munc13-1 (1:500, Synaptic Systems, Göttingen, Germany; Cat# 126113, RRID:AB_887734); rabbit anti-Munc13-2 (1:250; Synaptic Systems, Göttingen, Germany; Cat# 126203, RRID:AB_2619807, recognizing the brain-specific isoform, bMunc13-2), rabbit anti-mGluR7 (1:1,000; Millipore, MA, United States; Cat# 07-239, RRID: AB_310459), rabbit anti-Elfn2 (1:500, Sigma-Aldrich, MO, United States, Cat# HPA000781, RRID: AB_1079280) that recognizes both Elfn1 and Elfn 2 (Sylwestrak and Ghosh, 2012), rabbit anti-Elfn1 (1:500; Synaptic Systems, Göttingen, Germany; Cat#448-003, RRID:AB_2884915), guinea pig anti-mGluR7b (1:1,000 gift from Prof. Shigemoto, Shigemoto et al., 1997) diluted in TBS containing 2% NGS or NDS and 0.2% TritonX-100. After several washes, the following secondary antibodies were applied: Cy3-conjugated donkey anti-guinea pig (Cat#: 706-165-148, RRID: AB_2340460) or donkey anti-goat IgG (Cat#: 705-165-147, RRID: AB_2307351) and Cy5-conjugated donkey anti-rabbit IgG (Cat#711-175-152, RRID: AB_2340607; all from Jackson Immunoresearch Laboratories, PA, United States). Sections were either mounted in Vectashield or processed for multiplexed postembedding immunolabeling (see the section below). Image stacks were acquired with an Olympus FV1000 confocal microscope (Olympus Europa SE & Co., Hamburg, Germany) with 20x and 60x (oil immersion) objectives.

### Multiplexed Postembedding Immunolabeling

For details of this method (see Holderith et al., 2020). Briefly, sections following a preembedding immunoreaction (see the section above) for mGluR1α (1:1,000 visualized with Cy3-coupled donkey anti-goat Ab) and Munc13-2 (1:250; visualized with Cy5-coupled donkey anti-rabbit Ab) were washed several times in 0.1 M PB, dehydrated (without treatment with OsO_4_), and embedded into Durcupan. Preembedding immunolabeling of Munc13-2 was essential, because after epoxy embedding, our antibody did not recognize its epitope. 500 nm thick serial sections of the stratum oriens containing double immunolabeled IN dendrites were cut from the top 5 μm of the sections (to avoid heterogeneity from potential unequal penetration of the antibody into the tissue) and placed onto Superfrost Ultra plus slides. First, selected regions of interest (ROIs) were analyzed and imaged with Olympus FV1000 confocal microscope at high magnifications (60X objective; NA = 1.35). The resin was etched with Na-ethanolate (saturated solution of NaOH in absolute ethanol) for 5 min, then rinsed with 96% ethanol, and finally rinsed with distilled water. Retrieval of the proteins were carried out in 0.02 M Tris Base (pH = 9) containing 0.5% sodium dodecyl sulfate (SDS) at 80^°^C for 60 min. After several washes in TBS containing 0.05–0.1% Triton X-100 (TBST, pH = 7.6), the sections were blocked in TBST containing 6% Blotto A (Santa Cruz Biotechnology), 10% NGS, and 1% BSA (Sigma) for 30 min, then incubated in the guinea pig primary Ab against Munc13-1 (1:500, Synaptic Systems, Göttingen, Germany; Cat# 126104, RRID: AB_2619806) diluted in a blocking solution at room temperature, overnight. After several washes in TBST, the secondary Ab (Alexa 488 donkey anti-guinea pig, 1:200, Jackson ImmunoResearch Laboratories, PA, United States, Cat# 706-545-148, RRID: AB_2340472) was applied in TBST containing 10% of blocking solution for 2 h. After several washes in TBST, the slides were rinsed with distilled water, and the sections were mounted in SlowFade Diamond (Invitrogen, MA, United States). Images of ROIs were taken with an Olympus FV1000 confocal microscope and a 60x (oil immersion) objective or with an Abberior ExpertLine confocal microscope and a 100x (oil immersion) objective. Immunoglobulins were removed with a 5-min incubation in TBS containing 1% SDS (pH = 7.7) at 80^°^C. After 5 min of washing in TBST, new rounds of immunolabeling were performed using a guinea pig anti-PSD95 Ab (1:500, Synaptic Systems, Göttingen, Germany; Cat# 124014, RRID: AB_2619800), a guinea pig anti-panAMPA receptor Ab (1:100 Frontiers Cat#Af580; RRID: AB_257161), a chicken anti-Bassoon Ab (1:200 Synaptic Systems, Göttingen, Germany; Cat#141-016; RRID: AB_2661779), a guinea pig anti-Cav2.1 Ab (1:100 Synaptic Systems, Göttingen, Germany; Cat#152 205; RRID: AB_2619842), and finally with a guinea pig anti-Rim1/2 Ab (1:100 Synaptic Systems, Göttingen, Germany; Cat#140-205; RRID:AB_2631216) with the appropriate Alexa488-coupled secondary Abs (Alexa488-coupled donkey anti-guinea pig, 1:200; Jackson ImmunoResearch Laboratories, PA, United States; as above or Alexa488-coupled goat anti-chicken, 1:200; Jackson ImmunoResearch Laboratories, PA, United States, Cat# 103-545-155, RRID: AB_2337390).

### Quantitative Image Analysis

Confocal images were imported in ImageJ where circular ROIs (for postembedding immunolabeling) were placed over synaptic fluorescent clusters and over the unlabeled neuropil to determine the specific and non-specific labeling, respectively. The integral of the fluorescence was measured, and the background was subtracted for each ROI. For postembedding multiplexed labeling, images of the serial 500 nm thick sections were aligned using a custom-made module of ImageJ (“HyperStackStitcher;” available on the web site).^3^ To pool data from different experiments obtained with different confocal microscopes and laser settings, PSD95- normalized intensities of each immunosignal were further normalized to the population mean of the random synapses.

To quantify Munc13-2, Elfn, mGluR7, and mGluR1α immunolabeling intensity in Elfn KO and virally injected animals, freehand contour ROIs were used to outline mGluR1α+ dendrites in both the KO and the control or the injected and the contralateral hemispheres with same microscope settings and in the same depth of the tissue. The integral of the fluorescence was measured, and the background was subtracted for each ROI. About 40–150 ROIs were measured per animal.

### Statistical Analyses

Data are presented as mean ± SD and median throughout this study. All data were tested for normality using Shapiro–Wilk test. Non-normally distributed datasets were compared with Mann–Whitney *U*-test. Correlations were determined with Spearman’s rank correlation and the regression coefficient (r_*s*_); related *p*-values were calculated from two-tailed Student’s *t*-distribution. For multiple comparison, Kruskal–Wallis ANOVA was used with *post hoc* Dunn’s test. Statistically significant difference was assumed at *p* < 0.05.

### Materials

All the chemicals are obtained from Sigma, unless otherwise stated.

## Results

### Munc13-2 Is Selectively Present in Synapses-Innervating mGluR1α+ Dendrites

**FIGURE 1:**
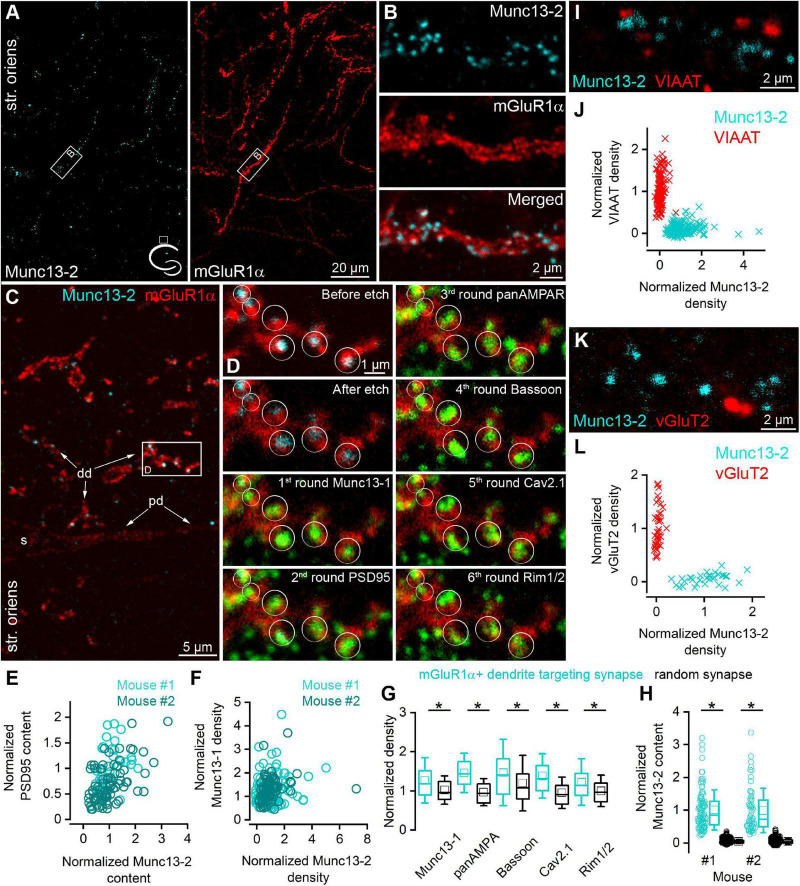
Munc13-2 immunolabeling is enriched on mGluR1α immunopositive dendrites. **(A)** Double immunolabeling for Munc13-2 (left, cyan) and mGluR1α (right, red) in the dorsal hippocampal CA1 region of the mouse (cartoon indicates the location of the region) shows similar distribution in the stratum oriens. Maximum intensity projection of six confocal images separated by 1 μm. **(B)** A dendritic segment of an mGluR1α immunopositive IN (white boxes on **A**) is shown at a higher magnification, which is decorated by Munc13-2 immunopositive puncta. Maximum intensity projection of three confocal images separated by 1 μm. **(C)** 500 nm thick epoxy resin embedded section with preembedding immunolabeling for Munc13-2 (cyan) and mGluR1α (red) shows that Munc13-2 immunopositive puncta preferentially located on the small diameter of mGluR1α+ (distal) dendrites (dd), and mainly avoid the soma (s) and a proximal dendrite (pd) in the hippocampus of the mouse. Boxed area is enlarged on panel **(D)**. **(D)** Multiplexed postembedding immunolabeling carried out on the section shown in panel **(G)**. Munc13-2 immunopositive puncta marked by circles (representing ROIs for quantification) along the mGluR1α immunolabeled dendrite are immunopositive for Munc13-1, PSD95, AMPA receptors, Bassoon, Cav2.1,and Rim1/2 (all pseudo colored to green). Note that the intensity of Munc13-2 immunolabeling varies substantially. Alignment of sections after each round was based on mGluR1α immunolabeling (red). Numbers represent the labeling rounds during the multiplexed labeling. **(E)** All of the Munc13-2 immunopositive puncta contain PSD95 immunosignal. Their amount shows positive correlations (Spearman correlation *r* = 0.48 and 0.55, *n* = 40 in mouse #1 and *n* = 80 in mouse #2). **(F)** Correlations between the density of the Munc13-2 and Munc13-1 in individual AZs (each data point represents an AZ, *n* = 114 in mouse #1 and *n* = 80 in mouse #2; Spearman correlation *r* = 0.16 and 0.34). **(G)** mGluR1α IN targeting synapses have significantly larger (*) Munc13-1, AMPA receptors, Bassoon, Cav2.1 and Rim1/2 densities than those found in randomly selected glutamatergic synapses in the str. oriens (*p* = 1.5 × 10^–5^, 5.5 × 10^–24^, 1.5 × 10^–4^, 6.6 × 10^–16^, 1.3 × 10^–5^, respectively, MW-*U*-test). Box plots represent median and 25/75 percentiles, square represent the mean value, whiskers represent SD. All immunolabelings were normalized to PSD95 intensity on panels **(F,G)**. **(H)** The Munc13-2 content of randomly selected synapses is only 4 ± 7% and 4 ± 10% (in two mice; *p* = 0 for both MW-*U*-test) of that of synapses on mGluR1α+ dendrites. **(I–L)** Munc13-2 immunolabeling (cyan) does not colocalize either with vesicular inhibitory amino acid transporter (VIAAT, red) (**I,J**, *n* = 152 Munc13-2 and 222 VIAAT positive profiles in 2 mice) or with vesicular glutamate transporter-2 (vGluT2, red) **(K,L)**, *n* = 43 Munc13-2 and 33 vGluT2 positive profiles in 1 mouse). Single confocal images **(I,K)**. str. oriens, stratum oriens.

Immunostaining of bMunc13-2 (brain specific isoform of the Munc13-2, referred as Munc13-2 in the study) in the dorsal hippocampus of adult mice [Figure 1, *n* = 3 C57BL6/J, *n* = 3 Tg(Chrna2-Cre)OE25Gsat/Mmucd, Leao et al., 2012] crossed with the reporter line Ai9 [Gt(ROSA)26Sor_CAG/LSL_tdTomato] and rats (*n* = 2, Supplementary Figure 1) revealed punctate labeling of neuronal processes in the stratum oriens and in the alveus of the CA1 area. Double immunofluorescent labeling of Munc13-2 and mGluR1α demonstrated that most of the Munc13-2 immunopositive puncta decorate mGluR1α+ dendrites and the majority of the mGluR1α+ dendrites are decorated by Munc13-2 puncta (Figures 1A,B). Since mGluR7 is present in presynaptic glutamatergic AZs that innervate mGluR1α+ INs (Shigemoto et al., 1996) and has a rather similar labeling pattern to that of Munc13-2, we performed colocalization of these two proteins. The majority of the mGluR7b puncta along the small diameter dendrites were Munc13-2 immunopositive and vice versa, and most Munc13-2 puncta were labeled for mGluR7b in rat (Supplementary Figures 1C,D). This suggested that Munc13-2 is present in excitatory synapses.

To further test the molecular composition of these Munc13-2 immunopositive synapses in mice, we applied postembedding multiplexed immunolabeling of a number of synaptic proteins on ultrathin sections obtained from epoxy resin-embedded tissue (Holderith et al., 2020) that had been immunolabeled for Munc13-2 and mGluR1α. We discovered that dehydration and resin embedding without OsO_4_ treatment retains the preembedding immunosignal when the reactions are visualized with Cy3- or Cy5-coupled secondary antibodies (Figure 1C). In 500 nm thick epoxy resin-embedded sections, Munc13-2 immunopositive puncta were clearly visible as they surrounded mGluR1α+ small diameter dendrites but spared the perisomatic and proximal dendritic membranes (Figure 1C). Following the removal of the resin with Na-ethanolate and antigen retrieval with an SDS solution, we immunolabeled the sections, first for Munc13-1, imaged the ROI, eluted the immunoglobulins, and relabeled the sections for PSD95, then for AMPA receptors, Bassoon, Cav2.1 voltage-gated Ca^2+^ channel (VGCC) subunit, and finally for Rim1/2 (Figure 1D). Qualitative assessment of the images revealed that the Munc13-2 positive puncta were immunopositive for all of these synaptic proteins, but to a different degree. We then quantified the fluorescent intensities for each protein in circular ROIs placed over the Munc13-2 positive puncta. We found that all Munc13-2 positive puncta contained PSD95 immunosignal (Figure 1E, *n* = 40 and 80 puncta in two mice) indicating that they are excitatory glutamatergic synapses. As the amount of PSD95 correlates almost perfectly with the size of the synapse (Cane et al., 2014; Karlocai et al., 2021), we normalized the immunosignal for each synaptic protein to that of PSD95 of the same synapse, resulting in density values that should be independent of the synapse size. To compare the data from different experiments, Munc13-2 density values were further normalized to the population mean of randomly selected synapses. Following this normalization, Munc13-2 density values displayed large variability (coefficient of variation: CV = 0.87 and 0.86 for mouse #1 and #2, respectively) among individual synapses and they did not correlate with normalized Munc13-1 density values (Figure 1F), suggesting that their amounts in the AZ are independently regulated. Interestingly, the PSD95 normalized densities of Munc13-1, AMPA receptors, Bassoon, Cav2.1, and Rim1/2 were significantly higher in synapses on mGluR1α+ dendrites compared to the randomly selected surrounding synapses (Munc13-1: 1.27 ± 0.58; AMPAR: 1.46 ± 0.50; Bassoon: 1.47 ± 0.85; Cav2.1: 1.38 ± 0.57; Rim1/2: 1.22 ± 0.6, *n* = 194 mGluR1α targeting and *n* = 160 random synapses from 2 mice; Figure 1G). To assess the selectivity of the Munc13-2 expression in mGluR1α+ dendrites targeting synapses, we measured their Munc13-2 content and compared them with that of randomly selected synapses in the surrounding neuropil in 2 mice (*n* = 101 and *n* = 60 mGluR1α+ dendrite targeting synapses, and *n* = 1,000 and *n* = 500 random synapses). Our quantification revealed that only 4 ± 6% and 4 ± 10% of the immunoreactivity found in mGluR1α+ dendrite targeting synapses are present in the surrounding randomly sampled synapses (Figure 1H).

Munc13-2 did not colocalize with vesicular inhibitory amino acid transporter (VIAAT; Figures 1I,J, *n* = 152 Munc13-2, and *n* = 222 VIAAT positive puncta in 2 mice, and Supplementary Figure 1F in 1 rat) or with vesicular glutamate transporter-2 (vGluT2; Figures 1K,L, *n* = 43 Munc13-2 and *n* = 33 vGluT2 positive puncta in 1 mouse, Supplementary Figure 1G in 1 rat). This indicates that most of the Munc13-2 labeled puncta are present on the axon terminals of local (CA1 and/or CA3) PCs.

### Lack of Munc13-2 Puncta in the Stratum Oriens of Elfn1 Knock-Out Mice

**FIGURE 2:**
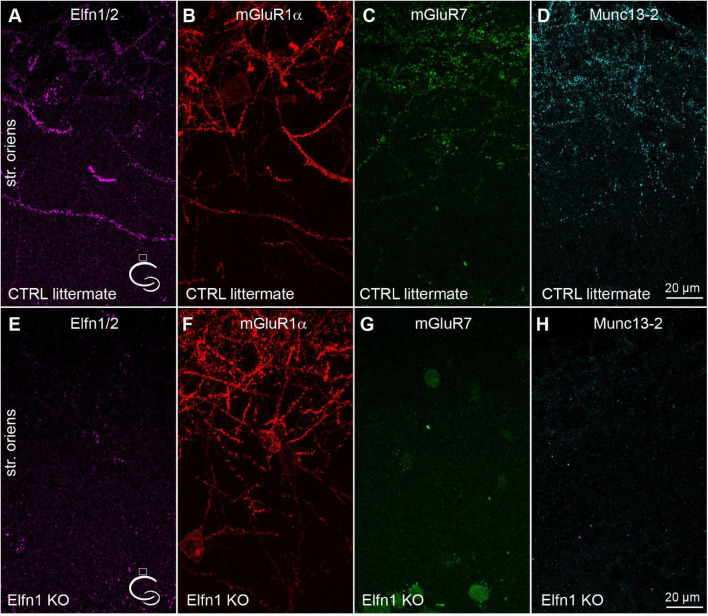
Munc13-2 and mGluR7 are missing in Elfn1 knock-out mice. **(A–D)** Immunolabeling for Elfn1/2 **(A)**, mGluR1α **(B)**, mGluR7 **(C)** and Munc13-2 **(D)** in the dorsal CA1 region of a littermate control mouse shows intense labeling of IN dendrites in the str. oriens. **(E–H)** same as **(A–D)** in an Elfn1 KO mouse. No specific immunolabeling is detected for Elfn1/2 **(E)**, mGluR7 **(G)** and Munc13-2 **(H)**. Cartoons indicate the location of the region. Maximum intensity projection of 20 confocal images separated by 1 μm. str. oriens, stratum oriens.

Elfn1 has been shown to be selectively expressed in Som/mGluR1α+ hippocampal INs (Sylwestrak and Ghosh, 2012), where it recruits mGluR7 to the presynaptic AZ (Tomioka et al., 2014; Stachniak et al., 2019). The selective knock-down of Elfn1 from these INs results in a decreased short-term facilitation. Next, we tested the potential role of Elfn1 in the above-described selective recruitment of Munc13-2. In five Elfn1 knock-out (KO) mice, immunolabeling of Elfn1/2 were clearly absent in the stratum oriens/alveus (Figures 2A,E). Quantitative measurement of Elfn1 immunolabeling showed a 97.7 ± 0.53% decrease in KO mice (*n* = 5) compared to the control littermates (*n* = 4) demonstrating that our antibody against Elfn1/2 provides specific labeling here for Elfn1 and also that the protein is missing in the KO mice. In line with previous results, we also found that immunolabeling of mGluR7 is dramatically reduced compared to the littermate heterozygous controls (Figures 2C,G) without any apparent change in the postsynaptic mGluR1α expression (Figures 2B,F, 107 ± 16% of control littermates, *n* = 5 KO mice, *n* = 4 control mice). Finally, we found that immunolabeling of Munc13-2 is apparently absent in the KO mice (3.7 ± 1.7% of control littermates; *n* = 5 KO mice and *n* = 4 control mice) compared to the littermate controls where punctate Munc13-2 immunolabeling is clearly visible along the mGluR1α+ dendrites (Figures 2D,H). The lack of both mGluR7 and Munc13-2 in Elfn1 KO mice raises the question whether the increased P_v_ at the PC-Som+ IN synapses found following Elfn1 knock-down is attributable to the lack of either mGluR7, or that of Munc13-2, or both.

### Conditional Knock-Out of Munc13-2 From CA1 Pyramidal Cells Does Not Affect mGluR7 Expression

**FIGURE 3:**
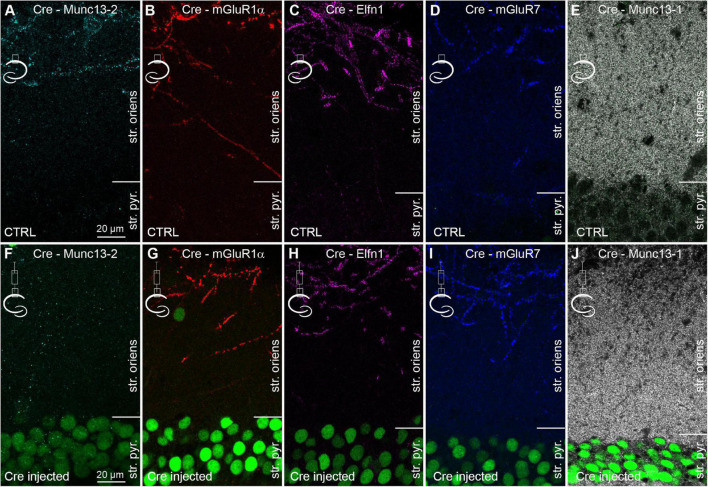
Conditional knock-out of Munc13-2 does not change the expression and distribution of Elfn1 and mGluR7. **(A–E)** Double immunolabeling for Cre and either Munc13-2 **(A)** or mGluR1α **(B)**, or Elfn1 **(C)** or mGluR7 **(D)** or Munc13-1 **(E)** in the dorsal CA1 area of the non-injected hemisphere. **(F–J)** Same as in **(A–E)**, but the images are from the hemisphere that has been injected with AAV expressing Cre-recombinase. Immunolabeled Cre (green) is visible in most CA1 PC nuclei (green). Note the lack of immunolabeling for Munc13-2 in the outer part of the stratum oriens, demonstrating the efficient removal of the protein, while there is no detectable change in the immunolabeling for mGluR1α, Elfn1, mGluR7, and Munc13-1. Maximum intensity projection of 4 confocal images separated by 1 μm. str. pyr, stratum pyramidale; str. oriens, stratum oriens.

To investigate the contribution of Munc13-2 to the properties of CA1 PC to mGluR1α+ IN synapses, Munc13-2 was conditionally knocked out from CA1 PCs in sixteen C57BL/6N-*Unc13b^TM 1a(KOMP)Wtsi^*/MbpMmucd mice with Cre-recombinase expressing AAVs injected unilaterally into the dorsal hippocampus (Figure 3). After 14 days, the mice were transcardially perfused and Cre expression was visualized with an anti-Cre antibody (Figure 3 and Supplementary Figure 2). In the non-injected hemisphere, no detectable anti-Cre immunoreactivity was observed in the nuclei of CA1 PCs and the immunolabeling patterns of Munc13-2, mGluR1α, Elfn1, mGluR7, and Munc13-1 (Figures 3A–E) were indistinguishable from those seen in the control mice and rats (Figure 1 and Supplementary Figure 1). In the central part of the injected area, the nuclei of apparently every PC were intensely labeled for Cre-recombinase and the mGluR1α+ dendrite associated specific immunosignal for Munc13-2 decreased by 92 ± 10% in the stratum oriens/alveus (*n* = 3 mice, Supplementary Figure 2G and Figure 3F). At the edges of the injection zone, the frequency of Cre-immunopositive nuclei decreased and Munc13-2 labeled structures emerged (Supplementary Figures 2A–C). In contrast, despite the expression of Cre and the lack of Munc13-2, the labeling patterns and the intensity of labeling for mGluR1α, Elfn1, mGluR7, and Munc13-1 were unchanged (Figures 3G–J and Supplementary Figure 2G, 101 ± 18%, 104 ± 5%, 104 ± 4%, 99 ± 1% of controls, respectively. *n* = 3 mice for mGluR1α, Elfn1, mGluR7, *n* = 2 mice for Munc13-1). In our injections, neither CA3 nor subicular PCs were transfected with Cre-expressing AAVs. Our results demonstrate that the majority of Munc13-2 immunosignal in the stratum oriens of the dorsal CA1 area originates from the axons of CA1 PCs and 2 weeks is sufficient for the significant removal of the protein from these synapses.

### Conditional Removal of Munc13-2 From CA1 Pyramidal Cells Leaves Unitary Excitatory Postsynaptic Currents in mGluR1α+ INs Unchanged

**FIGURE 4:**
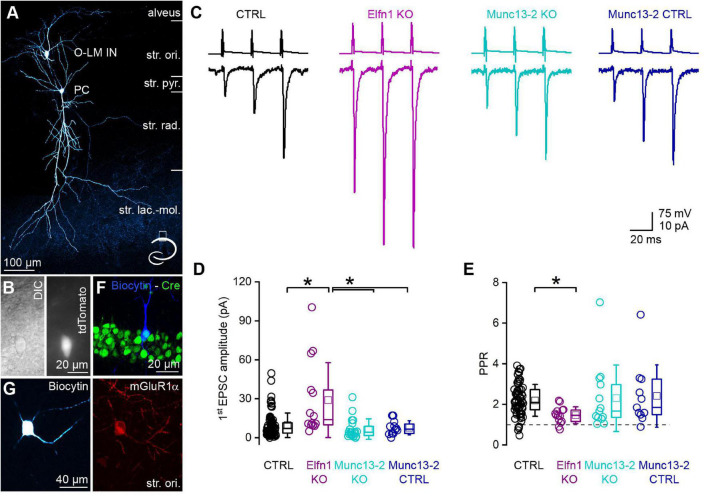
Removal of Munc13-2 does not change the peak amplitude and short-term plasticity of unitary EPSCs between CA1 PCs and mGluR1α expressing INs. **(A)** *In vitro* whole-cell patch-clamp recorded and biocytin filled CA1 PC and O-LM IN. The axonal arbor of the IN is visible in the stratum lacunosum-moleculare. Cartoon depicts the location of the cells within the hippocampus. **(B)** DIC image of the O-LM IN shown in panel **(A)** (left) and fluorescent tdTomato signal of the same cell (right). **(C)** Unitary EPSCs (lower traces) evoked by a train of 3 APs @ 40 Hz in a presynaptic CA1 PC (upper traces) recorded in postsynaptic O-LM INs in the dorsal CA1 region of the hippocampus. Black traces are from a control, magenta traces from an Elfn1 knock-out, cyan traces from a Munc13-2 conditional knock-out, dark blue traces from a Munc13-2 conditional knock-out littermate control. **(D)** The first EPSC is significantly larger (*) in the Elfn1 knock-out mouse than in any of the controls or in the Munc13-2 conditional knock-out mice (*p* = 5.44 × 10^–4^, Kruskal-Wallis ANOVA, *post hoc* Dunn’s test: *p* = 0.003, 0.00024, 0.041, ctrl vs. Elfn KO, Elfn KO vs. Munc13-2 KO, Elfn KO vs. Munc13-2 ctrl, respectively) while there is no change in the peak amplitude in the Munc13-2 conditional knock-out mouse compared to any of the controls (Kruskal–Wallis ANOVA, *post hoc* Dunn’s test *p* = 0.49 and 1, ctrl vs. Munc13-2 KO, control vs. Munc13-2 control). **(E)** The short-term facilitation is significantly less pronounced in the Elfn1 knock-out mouse (*p* = 0.0065, Kruskal-Wallis ANOVA, *post hoc* Dunn’s test: *p* = 0.0034, ctrl vs. Elfn knock-out) while there is no change in the short-term plasticity in the Munc13-2 conditional knock-out mouse compared to any of the controls (Kruskal-Wallis ANOVA, *post hoc* Dunn’s test, *p* = 1, ctrl vs. Munc13-2 KO, Munc13-2 KO vs. Munc13-2 control). **(F)** *In vitro* recorded and biocytin filled PC (blue) expressing Cre-recombinase (green) that is localized to the PC nucleus. Single confocal image. **(G)** A biocytin filled IN with truncated axon (left) expresses mGluR1α (right red). Maximum intensity projection of four confocal images separated by 1 μm. Box plots represent median and 25/75 percentiles, square represent the mean value, whiskers represent SD. str. ori., stratum oriens, str. pyr., stratum pyramidale, str. rad., stratum radiatum, str. lac.-mol., stratum lacunosum-moleculare.

Postsynaptic responses between CA1 PCs and mGluR1α+ INs have small amplitudes and display marked short-term facilitation. We recorded from a large population of connected CA1 PC to mGluR1α+ IN pairs in a transgenic mouse line that expresses the red fluorescent protein, tdTomato in O-LM INs in the stratum oriens of the CA1 region (Figures 4A,B). This allowed us to efficiently select mGluR1α+ /O-LM INs (50 out of 52 *post hoc* identified INs had O-LM morphology, and only 2 were bistratified cells, Figure 4A). We recorded unitary excitatory postsynaptic currents (uEPSCs) evoked by a short train of presynaptic action potentials (APs) at 40 Hz (Figure 4C, black). The first uEPSC had a small amplitude (9.6 ± 9.4 pA, median = 7.2 pA, *n* = 80 pairs in 44 mice; Figure 4D, black) and the paired pulse ratio (PPR, 2nd uEPSC/1st uEPSC) was 2.19 ± 0.78 (median = 2.09, *n* = 66 in 38 animals, in 14 cell pairs, the 1st uEPSC peak amplitude was 0 pA, precluding the calculation of the PPR, Figure 4E, black). The amplitude of the 3rd uEPSC increased further resulting in a third uEPSC/first uEPSC ratio of 2.99 ± 1.25 (median = 2.74, *n* = 66 in 38 mice). In Elfn1 KO mice, the amplitude of the first uEPSC was significantly larger (29.0 ± 28.9 pA, median = 13.9 pA, *n* = 14 pairs in 10 mice; Figures 4C,D magenta) than that in the control mice. In these KO animals, presumed mGluR1α+ /O-LM INs were preselected based on the location, size, shape of their somata, and on their firing patterns upon DC current injections. Following the recordings, we characterized the cells *post hoc* and found that 10 out of 14 cells had O-LM morphology and the remaining 4 cells had truncated axon, but they were immunopositive for mGluR1α. The 3-times increase in the first uEPSC is accompanied by a significant decrease of the PPR (1.46 ± 0.41, *n* = 14 pairs in 10 mice; Figure 4E). As discussed above, the altered uEPSCs in Elfn1 KO mice could be the consequence of the loss of mGluR7 and/or Munc13-2. To address the contribution of Munc13-2, we performed paired recordings between CA1 PCs and presumed mGluR1α+ /O-LM INs in slices from conditional Munc13-2 KO mice in which the dorsal CA1 region was injected with Cre- and mCherry-expressing AAVs. In these animals, PCs were selected based on the expression of mCherry, and the presence of Cre-recombinase in the recorded PCs was verified *post hoc* by immunolabeling for Cre. Only those pairs were included where the Cre immunopositivity was unequivocally identified in the presynaptic PC (Figure 4F). Similar to the Elfn1 KO mice, in this transgenic line, mGluR1α+ /O-LM INs were selected in the slice based on the same criteria as described above and were molecularly and morphologically characterized *post hoc* (10 out of 20 cells were O-LM cells, 10 out of 20 cells have truncated axons, but they were immunopositive for mGluR1α; Figure 4G). The first uEPSC of the short train had a small amplitude (6.7 ± 7.9 pA, median = 4.3 pA, *n* = 20 pairs in 13 animals) which was not significantly different from that recorded in the control mice (Figure 4D). The amplitudes of the second (14.0 ± 13.7 pA, median = 7.1 pA) and the third uEPSCs (18.7 ± 19.9 pA, median = 7.6 pA) were also similar to those in the control, resulting in a PPR of 2.30 ± 1.64 (median = 1.66, *n* = 13 pairs in 10 mice, for 7 cell pairs the amplitude of the 1st uEPSC peak was 0 pA, precluding the calculation of the PPR; Figure 2E), which was not significantly different from that in the control. Since the Munc13-2 KO mice had a different genetic background (C57BL/6N) compared to the control mice (C57BL/6J), we recorded from the contralateral, non-injected hemisphere of Munc13-2 conditional KO mice as an independent control population and found that the amplitude (first: 7.7 ± 5.5 pA, median = 6.7 pA; second: 17.0 ± 11.8 pA, median = 17.3 pA; third: 27.1 ± 18.6 pA, median = 27.1 pA, *n* = 12 pairs in 6 mice, Figures 4C,D) and PPR (2.4 ± 1.5; Figure 4E) of uEPSCs were not significantly different from those recorded from the control or the Munc13-2 KO mice.

## Discussion

In the present manuscript, we describe that: (1) bMunc13-2 is selectively enriched in AZs of CA1 PC axon terminals that target mGluR1α+ INs (mainly O-LM INs). (2) Munc13-1 is also present in Munc13-2 containing CA1 PC AZs, together with Rim1/2, Cav2.1, and Bassoon. (3) Elfn1 is expressed by the postsynaptic mGluR1α+ INs and trans-synaptically recruits not only mGluR7, but also Munc13-2 into AZs. (4) Conditional genetic deletion of Munc13-2 does not change the distribution of either Elfn1 or its presynaptic signaling partner, mGluR7. (5) CA1 PC to mGluR1α+ /O-LM IN synapses lacking Munc13-2 display very similar functional properties compared to Munc13-2 containing control synapses.

Postsynaptic responses made by hippocampal PCs onto mGluR1α+ /O-LM INs display marked short-term facilitation upon repetitive stimulation (Ali and Thomson, 1998; Biro et al., 2005). Despite several studies aiming to reveal the mechanisms of how facilitation is manifested at this synapse, the key molecular mechanism remains still elusive. A selective enrichment of mGluR7 in these presynaptic AZs had been described over two decades ago (Shigemoto et al., 1996) and the contribution of constitutively active group III mGluRs (including mGluR7) to the small EPSC amplitude has been reported (Losonczy et al., 2003). However, neither pharmacological block nor genetic manipulations that remove mGluR7 increased the EPSC amplitude markedly enough at this synapse, leaving the characteristic short-term facilitation intact (Sylwestrak and Ghosh, 2012; Tomioka et al., 2014; Stachniak et al., 2019). A previous study from our laboratory (Eltes et al., 2017) and the study by Koester and Johnston (2005) described smaller [Ca^2+^] transients in the PC terminals synapsing on Som/mGluR1α+ dendrites compared to those that synapse PV+ INs. However, this difference in [Ca^2+^] is unlikely to be responsible for the low P_v_ at this synapse, because the application of 4-aminopyridin elevated the presynaptic [Ca^2+^] in boutons innervating mGluR1α+ INs to reach that found in boutons innervating PV expressing INs, but the EPSCs, though increased, still remained small and facilitating at PC to mGluR1α+ IN synapses (Holderith et al., unpublished observation). A longer coupling distance between SVs and presynaptic VGCCs was also suggested as a potential mechanism of the low P_v_ at cortical PC to Som+ IN synapses (Rozov et al., 2001), but freeze-fracture replica immunolabeling experiments could not confirm different nano-topologies at the hippocampal PC to mGluR1α+ vs. PC to PV+ IN synapses (Lorincz et al., unpublished observation), suggesting that a different coupling distance as a key mechanism is also unlikely.

Since none of the above-mentioned mechanisms seems to be key in setting the P_v_ exceptionally low at PC to Som/mGluR1α+ IN synapses, we tested the subcellular distribution of presynaptic proteins with critical roles in SV docking/priming and tried to find whether any of them has a postsynaptic cell type-dependent distribution in PC axons. Munc13-2 showed a punctate labeling in the stratum oriens of the dorsal hippocampal CA1 area, and our colocalization experiments revealed that it is selectively enriched in the AZs of local CA1 PC boutons that innervate mGluR1α+ INs. To our knowledge, this is the third example (mGluR7: Shigemoto et al., 1996 and mGluR8: Ferraguti et al., 2005) of such postsynaptic target cell type-specific distribution of presynaptic molecules in cortical networks, suggesting that this phenomenon might be more common than previously envisaged. Munc13s are essential synaptic vesicle priming factors and are indispensable for synaptic vesicle fusion (Augustin et al., 1999b; Varoqueaux et al., 2002; Sudhof and Rizo, 2011). They form a tripartite complex with RIMs and RIM-binding proteins that collaborate to recruit VGCCs and SVs to the AZ (Wang et al., 1997; Betz et al., 2001; Schoch et al., 2002; Andrews-Zwilling et al., 2006; Kaeser et al., 2011; Brockmann et al., 2019, 2020). Out of the three isoforms expressed in the CNS (Munc13-1 to Munc13-3), Munc13-1 has the broadest distribution, while Munc13-2 and 3 have more restricted and largely non-overlapping expression patterns (Augustin et al., 1999a). Several studies investigated the functional roles of Munc13s in the heterologous expression systems or neuronal cultures (Rhee et al., 2002; Rosenmund et al., 2002; Varoqueaux et al., 2002; Van De Bospoort et al., 2012). For example, Rosenmund et al. (2002) demonstrated that in cultured hippocampal neurons, Munc13-1 is likely to contribute to the tight docking of vesicles and confer high P_v_, whereas in Munc13-2 containing AZs, SVs are loosely docked and have low P_v_. Based on these results and the preferential location of Munc13-2 in AZs innervating mGluR1α+ INs, we hypothesized that this Munc isoform will have a key role in setting the P_v_ low in this synapse. However, to our surprise, the conditional removal of Munc13-2 from CA1 PCs (in an intact neuronal network) had no apparent effect on the P_v_ of the CA1 PC to mGluR1α+ IN synapses. Similar results were found at mouse photoreceptor ribbon synapses, where Munc13-2 is the only Munc13 isoform present (Cooper et al., 2012), although this synapse differs from the conventional synapses in many ways. A similar lack of effect was found however at Schaffer collateral input onto CA1 PCs (Breustedt et al., 2010) and in the calyx of Held (Chen et al., 2013) in Munc13-2 KO animals. Although, it should be noted that the amount of bMunc13-2 is low in Schaffer collateral to CA1 PC synapses in control animals, therefore the lack of effect in the KO is not that surprising. Furthermore, in the same Munc13-2 KO animals, an apparent reduction of P_v_ was found at the hippocampal mossy fiber synapses onto CA3 PCs (Breustedt et al., 2010). Munc13-2 knock down with shRNA from glutamatergic input onto amygdala PCs increases P_v_ (Gioia et al., 2016), indicating a complex role of Munc13-2 in SV priming, probably depending on its interactive partners in the protein complex of the AZ. Here we show that CA1 PC to mGluR1α+ IN synapses also contain Munc13-1, which requires Rim1/2 and Rim binding protein for the efficient priming activity (Deng et al., 2011; Brockmann et al., 2020). Thus, the lack of Rim1/2 at this synapse could provide an explanation for the low P_v_. We directly tested this hypothesis using multiplexed immunolabeling of Munc13-2 positive synapses on mGluR1α+ INs and found that Rim1/2 has an even higher density in this synapse compared to that found in the surrounding synapses that are likely to be on PC spines. Thus, we conclude that Munc13-1 must have additional interactive partners or regulatory pathways that adjust its efficacy in priming SVs in a target cell type-dependent manner.

Although, knocking down the expression of Elfn1 with shRNA did not turn EPSCs on Som+ INs depressing there was a reduction in the paired pulse facilitation, indicating some change in the P_v_ of the synapse (Sylwestrak and Ghosh, 2012), which was later attributed to the lack of presynaptic mGluR7 (Tomioka et al., 2014; Stachniak et al., 2019). Here, we tested how the expression of the specifically located Munc13-2 in the AZs innervating mGluR1α+ INs is altered in the Elfn1 KO mice. Our results revealed that Munc13-2 is enriched in CA1 PC to mGluR1α+ IN synapses in an Elfn1-dependent manner. Removal of Elfn1 results in the loss of mGluR7 and Munc13-2 and a threefold increase in the peak amplitude of PC to mGluR1α+ /O-LM IN uEPSCs and a decreased short-term facilitation. To distinguish whether the functional effect is due to the lack of constitutive mGluR7 activity or lack of Munc13-2, we conditionally removed Munc13-2 (both bMunc13-2 and ubMunc13-2 isoforms) from these synapses by injecting Cre-recombinase expressing AAVs into the dorsal hippocampal CA1 area of the transgenic mouse line in which the Munc13-2 exons, 15–17 are placed between two loxP sites. As no effect of Munc13-2 removal was found on uEPSC amplitudes and PPRs, we conclude that the functional effects of removing Elfn1 from Som/mGluR1α+ INs are the sole consequence of the lack of mGluR7. This is in line with the results of pharmacological block of group III mGluRs that has a very similar effect in EPSC amplitudes (Losonczy et al., 2003).

In summary, although the hippocampal CA1 PC to mGluR1α+ /O-LM IN synapses contain Munc13-2 at high concentration, it does not play a role in setting the P_v_ unusually low, indicating that Munc13-1 is capable of “ill-priming” SVs or there are additional molecules that prevent tightly docked vesicles from being released, the identity of which is to be discovered.

## Data Availability Statement

The raw data supporting the conclusions of this article will be made available by the authors, without undue reservation.

## Ethics Statement

The animal study was reviewed and approved by the Animal Committee of the Institute of Experimental Medicine, Budapest.

## Author Contributions

NH and ZN designed the experiments and wrote the manuscript. MA performed the *in vitro* paired recording and analyzed the data. NH performed some paired recordings and all immunolocalization experiments and analyzed the data. All authors contributed to the article and approved the submitted version.

## Conflict of Interest

The authors declare that the research was conducted in the absence of any commercial or financial relationships that could be construed as a potential conflict of interest.

## Publisher’s Note

All claims expressed in this article are solely those of the authors and do not necessarily represent those of their affiliated organizations, or those of the publisher, the editors and the reviewers. Any product that may be evaluated in this article, or claim that may be made by its manufacturer, is not guaranteed or endorsed by the publisher.

## Footnotes

1
http://n2t.net/addgene:55632

2
http://n2t.net/addgene:105558

3
http://nusserlab.hu/software.html

## References

[B1] Ali A. B. Thomson A. M. (1997). Brief train depression and facilitation at pyramidal-interneurone connections in slices of rat hippocampus; paired recordings with biocytin filling. *J. Physiol.* 9:501.

[B2] Ali A. B. Thomson A. M. (1998). Facilitating pyramid to horizontal oriens-alveus interneurone inputs: dual intracellular recordings in slices of rat hippocampus. *J. Physiol.* 507 185–199. 10.1111/j.1469-7793.1998.185bu.x 9490837 PMC2230767

[B3] Andrews-Zwilling Y. S. Kawabe H. Reim K. Varoqueaux F. Brose N. (2006). Binding to Rab3A-interacting molecule RIM regulates the presynaptic recruitment of Munc13-1 and ubMunc13-2. *J. Biol. Chem.* 281 19720–19731. 10.1074/jbc.M601421200 16704978

[B4] Augustin I. Betz A. Herrmann C. Jo T. Brose N. (1999a). Differential expression of two novel Munc13 proteins in rat brain. *Biochem. J.* 337 363–371. 9895278 PMC1219986

[B5] Augustin I. Rosenmund C. Sudhof T. C. Brose N. (1999b). Munc13-1 is essential for fusion competence of glutamatergic synaptic vesicles. *Nature* 400 457–461. 10.1038/22768 10440375

[B6] Basu J. Shen N. Dulubova I. Lu J. Guan R. Guryev O. (2005). A minimal domain responsible for Munc13 activity. *Nat. Struct. Mol. Biol.* 12 1017–1018. 10.1038/nsmb1001 16228007

[B7] Betz A. Thakur P. Junge H. J. Ashery U. Rhee J. S. Scheuss V. (2001). Functional interaction of the active zone proteins Munc13-1 and RIM1 in synaptic vesicle priming. *Neuron* 30 183–196. 10.1016/s0896-6273(01)00272-0 11343654

[B8] Biro A. A. Holderith N. B. Nusser Z. (2005). Quantal size is independent of the release probability at hippocampal excitatory synapses. *J. Neurosci.* 25 223–232. 10.1523/JNEUROSCI.3688-04.2005 15634785 PMC6725207

[B9] Breustedt J. Gundlfinger A. Varoqueaux F. Reim K. Brose N. Schmitz D. (2010). Munc13-2 differentially affects hippocampal synaptic transmission and plasticity. *Cereb. Cortex* 20 1109–1120. 10.1093/cercor/bhp170 19700493

[B10] Brockmann M. M. Maglione M. Willmes C. G. Stumpf A. Bouazza B. A. Velasquez L. M. (2019). RIM-BP2 primes synaptic vesicles via recruitment of Munc13-1 at hippocampal mossy fiber synapses. *Elife* 8:e43243. 10.7554/eLife.43243 31535974 PMC6752948

[B11] Brockmann M. M. Zarebidaki F. Camacho M. Grauel M. K. Trimbuch T. Sudhof T. C. (2020). A Trio of Active Zone Proteins Comprised of RIM-BPs. *Cell Rep.* 32:107960. 10.1016/j.celrep.2020.107960 32755572

[B12] Brose N. Hofmann K. Hata Y. Sudhof T. C. (1995). Mammalian homologues of Caenorhabditis elegans unc-13 gene define novel family of C2-domain proteins. *J. Biol. Chem.* 270 25273–25280. 10.1074/jbc.270.42.25273 7559667

[B13] Cane M. Maco B. Knott G. Holtmaat A. (2014). The relationship between PSD-95 clustering and spine stability *in vivo*. *J. Neurosci.* 34 2075–2086. 10.1523/JNEUROSCI.3353-13.2014 24501349 PMC6608529

[B14] Chen Z. Cooper B. Kalla S. Varoqueaux F. Young S. M. Jr. (2013). The Munc13 proteins differentially regulate readily releasable pool dynamics and calcium-dependent recovery at a central synapse. *J. Neurosci.* 33 8336–8351. 10.1523/JNEUROSCI.5128-12.2013 23658173 PMC6619620

[B15] Cooper B. Hemmerlein M. Ammermuller J. Imig C. Reim K. Lipstein N. (2012). Munc13-independent vesicle priming at mouse photoreceptor ribbon synapses. *J. Neurosci.* 32 8040–8052. 10.1523/JNEUROSCI.4240-11.2012 22674279 PMC6620942

[B16] Deng L. Kaeser P. S. Xu W. Sudhof T. C. (2011). RIM proteins activate vesicle priming by reversing autoinhibitory homodimerization of Munc13. *Neuron* 69 317–331. 10.1016/j.neuron.2011.01.005 21262469 PMC3063404

[B17] Eltes T. Kirizs T. Nusser Z. Holderith N. (2017). Target Cell Type-Dependent Differences in Ca2 + Channel Function Underlie Distinct Release Probabilities at Hippocampal Glutamatergic Terminals. *J. Neurosci.* 37 1910–1924. 10.1523/JNEUROSCI.2024-16.2017 28115484 PMC6589975

[B18] Fenno L. E. Mattis J. Ramakrishnan C. Hyun M. Lee S. Y. He M. (2014). Targeting cells with single vectors using multiple-feature Boolean logic. *Nat. Methods* 11 763–772. 10.1038/nmeth.2996 24908100 PMC4085277

[B19] Ferraguti F. Klausberger T. Cobden P. Baude A. Roberts J. D. Szucs P. (2005). Metabotropic glutamate receptor 8-expressing nerve terminals target subsets of GABAergic neurons in the hippocampus. *J. Neurosci.* 25 10520–10536. 10.1523/JNEUROSCI.2547-05.2005 16280590 PMC6725819

[B20] Gioia D. A. Alexander N. J. Mccool B. A. (2016). Differential Expression of Munc13-2 Produces Unique Synaptic Phenotypes in the Basolateral Amygdala of C57BL/6J and DBA/2J Mice. *J. Neurosci.* 36 10964–10977. 10.1523/JNEUROSCI.1785-16.2016 27798178 PMC5098836

[B21] Holderith N. Heredi J. Kis V. Nusser Z. (2020). A High-Resolution Method for Quantitative Molecular Analysis of Functionally Characterized Individual Synapses. *Cell Rep.* 32:107968. 10.1016/j.celrep.2020.107968 32726631 PMC7408500

[B22] Kaeser P. S. Deng L. Wang Y. Dulubova I. Liu X. Rizo J. (2011). RIM proteins tether Ca2 + channels to presynaptic active zones via a direct PDZ-domain interaction. *Cell* 144 282–295. 10.1016/j.cell.2010.12.029 21241895 PMC3063406

[B23] Karlocai M. R. Heredi J. Benedek T. Holderith N. Lorincz A. Nusser Z. (2021). Variability in the Munc13-1 content of excitatory release sites. *Elife* 10:e67468. 10.7554/eLife.67468 33904397 PMC8116053

[B24] Kawabe H. Mitkovski M. Kaeser P. S. Hirrlinger J. Opazo F. Nestvogel D. (2017). ELKS1 localizes the synaptic vesicle priming protein bMunc13-2 to a specific subset of active zones. *J. Cell Biol.* 216 1143–1161. 10.1083/jcb.201606086 28264913 PMC5379939

[B25] Koester H. J. Johnston D. (2005). Target cell-dependent normalization of transmitter release at neocortical synapses. *Science* 308 863–866. 10.1126/science.1100815 15774725

[B26] Leao R. N. Mikulovic S. Leao K. E. Munguba H. Gezelius H. Enjin A. (2012). OLM interneurons differentially modulate CA3 and entorhinal inputs to hippocampal CA1 neurons. *Nat. Neurosci.* 15 1524–1530. 10.1038/nn.3235 23042082 PMC3483451

[B27] Li W. Ma C. Guan R. Xu Y. Tomchick D. R. Rizo J. (2011). The crystal structure of a Munc13 C-terminal module exhibits a remarkable similarity to vesicle tethering factors. *Structure* 19 1443–1455. 10.1016/j.str.2011.07.012 22000513 PMC3197213

[B28] Losonczy A. Somogyi P. Nusser Z. (2003). Reduction of excitatory postsynaptic responses by persistently active metabotropic glutamate receptors in the hippocampus. *J. Neurophysiol.* 89 1910–1919. 10.1152/jn.00842.2002 12686572

[B29] Man K. N. Imig C. Walter A. M. Pinheiro P. S. Stevens D. R. Rettig J. (2015). Identification of a Munc13-sensitive step in chromaffin cell large dense-core vesicle exocytosis. *Elife* 4:e10635. 10.7554/eLife.10635 26575293 PMC4798968

[B30] Neher E. Brose N. (2018). Dynamically Primed Synaptic Vesicle States: key to Understand Synaptic Short-Term Plasticity. *Neuron* 100 1283–1291. 10.1016/j.neuron.2018.11.024 30571941

[B31] Pouille F. Scanziani M. (2004). Routing of spike series by dynamic circuits in the hippocampus. *Nature* 429 717–723. 10.1038/nature02615 15170216

[B32] Reyes A. Lujan R. Rozov A. Burnashev N. Somogyi P. Sakmann B. (1998). Target-cell-specific facilitation and depression in neocortical circuits. *Nat. Neurosci.* 1 279–285. 10.1038/1092 10195160

[B33] Rhee J. S. Betz A. Pyott S. Reim K. Varoqueaux F. Augustin I. (2002). Beta phorbol ester- and diacylglycerol-induced augmentation of transmitter release is mediated by Munc13s and not by PKCs. *Cell* 108 121–133. 10.1016/s0092-8674(01)00635-3 11792326

[B34] Rosenmund C. Sigler A. Augustin I. Reim K. Brose N. Rhee J. S. (2002). Differential control of vesicle priming and short-term plasticity by Munc13 isoforms. *Neuron* 33 411–424. 10.1016/s0896-6273(02)00568-8 11832228

[B35] Rozov A. Burnashev N. Sakmann B. Neher E. (2001). Transmitter release modulation by intracellular Ca2 + buffers in facilitating and depressing nerve terminals of pyramidal cells in layer 2/3 of the rat neocortex indicates a target cell-specific difference in presynaptic calcium dynamics. *J. Physiol.* 531 807–826. 10.1111/j.1469-7793.2001.0807h.x 11251060 PMC2278500

[B36] Sakamoto H. Ariyoshi T. Kimpara N. Sugao K. Taiko I. Takikawa K. (2018). Synaptic weight set by Munc13-1 supramolecular assemblies. *Nat. Neurosci.* 21 41–49. 10.1038/s41593-017-0041-9 29230050

[B37] Scanziani M. Gahwiler B. H. Charpak S. (1998). Target cell-specific modulation of transmitter release at terminals from a single axon. *Proc. Natl. Acad. Sci. U.S.A.* 95 12004–12009. 10.1073/pnas.95.20.12004 9751780 PMC21755

[B38] Schoch S. Castillo P. E. Jo T. Mukherjee K. Geppert M. Wang Y. (2002). RIM1alpha forms a protein scaffold for regulating neurotransmitter release at the active zone. *Nature* 415 321–326. 10.1038/415321a 11797009

[B39] Shigemoto R. Kinoshita A. Wada E. Nomura S. Ohishi H. Takada M. (1997). Differential presynaptic localization of metabotropic glutamate receptor subtypes in the rat hippocampus. *J. Neurosci.* 17 7503–7522. 10.1523/JNEUROSCI.17-19-07503.1997 9295396 PMC6573434

[B40] Shigemoto R. Kulik A. Roberts J. D. Ohishi H. Nusser Z. Kaneko T. (1996). Target-cell-specific concentration of a metabotropic glutamate receptor in the presynaptic active zone. *Nature* 381 523–525. 10.1038/381523a0 8632825

[B41] Siksou L. Rostaing P. Lechaire J. P. Boudier T. Ohtsuka T. Fejtova A. (2007). Three-dimensional architecture of presynaptic terminal cytomatrix. *J. Neurosci.* 27 6868–6877. 10.1523/JNEUROSCI.1773-07.2007 17596435 PMC6672225

[B42] Stachniak T. J. Sylwestrak E. L. Scheiffele P. Hall B. J. Ghosh A. (2019). Elfn1-Induced Constitutive Activation of mGluR7 Determines Frequency-Dependent Recruitment of Somatostatin Interneurons. *J. Neurosci.* 39 4461–4474. 10.1523/JNEUROSCI.2276-18.2019 30940718 PMC6554623

[B43] Stevens D. R. Wu Z. X. Matti U. Junge H. J. Schirra C. Becherer U. (2005). Identification of the minimal protein domain required for priming activity of Munc13-1. *Curr. Biol.* 15 2243–2248. 10.1016/j.cub.2005.10.055 16271475

[B44] Sudhof T. C. (2012). The presynaptic active zone. *Neuron* 75 11–25. 10.1016/j.neuron.2012.06.012 22794257 PMC3743085

[B45] Sudhof T. C. Rizo J. (2011). Synaptic vesicle exocytosis. *Cold Spring Harb. Perspect. Biol.* 3:a005637. 10.1101/cshperspect.a005637 PMC3225952 22026965

[B46] Sun H. Y. Lyons S. A. Dobrunz L. E. (2005). Mechanisms of target-cell specific short-term plasticity at Schaffer collateral synapses onto interneurones versus pyramidal cells in juvenile rats. *J. Physiol.* 568 815–840. 10.1113/jphysiol.2005.093948 16109728 PMC1464188

[B47] Sylwestrak E. L. Ghosh A. (2012). Elfn1 regulates target-specific release probability at CA1-interneuron synapses. *Science* 338 536–540. 10.1126/science.1222482 23042292 PMC5297939

[B48] Tomioka N. H. Yasuda H. Miyamoto H. Hatayama M. Morimura N. Matsumoto Y. (2014). Elfn1 recruits presynaptic mGluR7 in trans and its loss results in seizures. *Nat. Commun.* 5:4501. 10.1038/ncomms5501 25047565

[B49] Van De Bospoort R. Farina M. Schmitz S. K. De Jong A. De Wit H. Verhage M. (2012). Munc13 controls the location and efficiency of dense-core vesicle release in neurons. *J. Cell Biol.* 199 883–891. 10.1083/jcb.201208024 23229896 PMC3518216

[B50] Varoqueaux F. Sigler A. Rhee J. S. Brose N. Enk C. Reim K. (2002). Total arrest of spontaneous and evoked synaptic transmission but normal synaptogenesis in the absence of Munc13-mediated vesicle priming. *Proc. Natl. Acad. Sci. U.S.A.* 99 9037–9042. 10.1073/pnas.122623799 12070347 PMC124419

[B51] Wang Y. Okamoto M. Schmitz F. Hofmann K. Sudhof T. C. (1997). Rim is a putative Rab3 effector in regulating synaptic-vesicle fusion. *Nature* 388 593–598. 10.1038/41580 9252191

