# Supplementary material for: Selective Enrichment of Munc13-2 in Presynaptic Active Zones of Hippocampal Pyramidal Cells That Innervate mGluR1α Expressing Interneurons
Source: Front Synaptic Neurosci. 2022 Feb 10;13:773209. doi: 10.3389/fnsyn.2021.773209 (PMC8866005; doi:10.3389/fnsyn.2021.773209)
Supplement: Supplementary file 1 [file Data_Sheet_1.PDF]

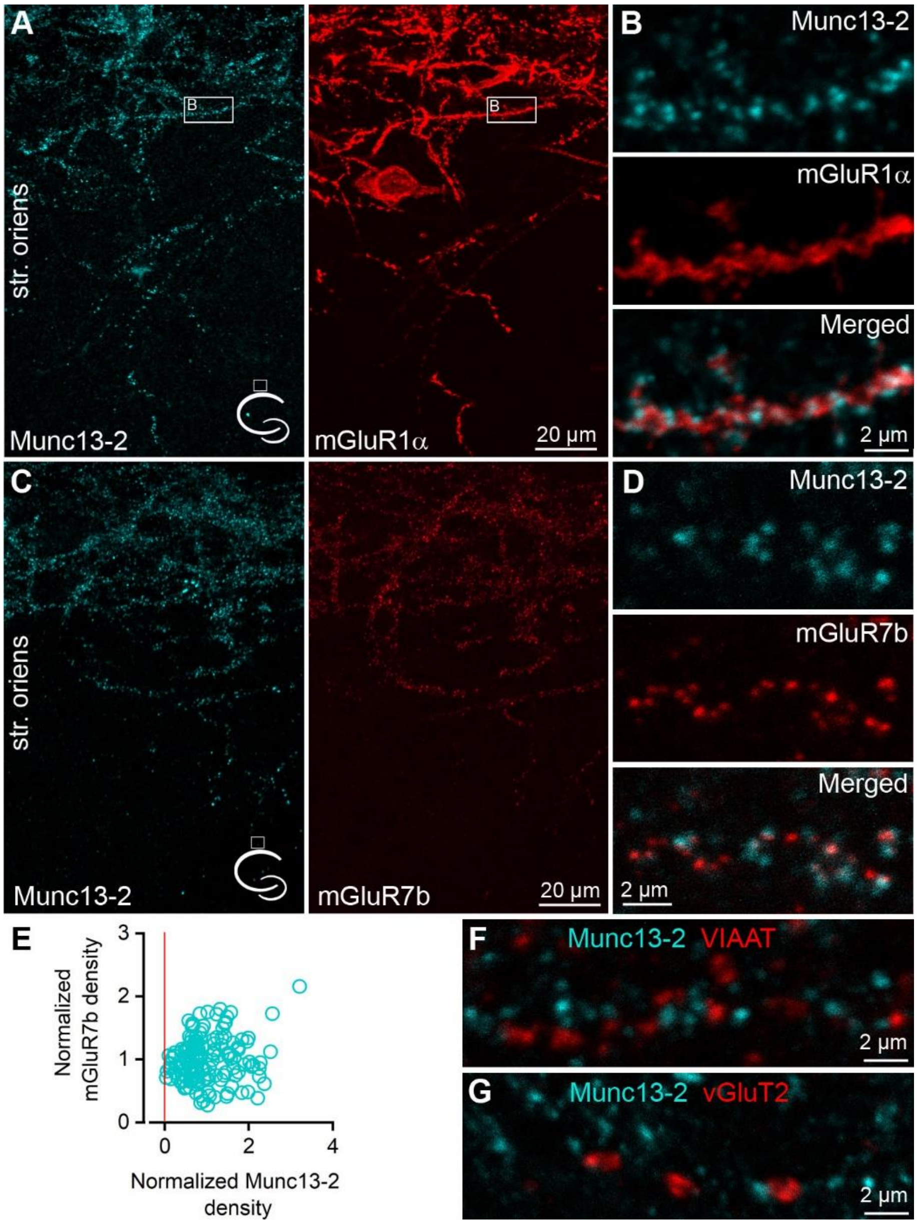

Supplementary Figure 1.

Munc13-2 immunolabeling is enriched on mGluR1α immunopositive dendrites. (**A**) Immunolabeling for Munc13-2 (left, cyan) and mGluR1α (right, red) shows similar distribution in the stratum oriens in the dorsal hippocampal CA1 region of a rat (cartoon indicates the location of the region). Maximum intensity projection of 20 confocal images separated by 1 µm. (**B**) A dendritic segment of an mGluR1α immunopositive IN (white boxes on panel A) is shown at a higher magnification, which is decorated by Munc13-2 immunopositive puncta. Maximum intensity projection of 2 confocal images separated by 0.5 µm. (**C**) Same as in A but for Munc13-2 (left, cyan) and mGluR7b (right, red). (**D**) Munc13-2 and mGluR7b positive puncta show prominent colocalization. Maximum intensity projection of 2 confocal images separated by 0.5 µm. (**E**) All of Munc13-2 immunopositive puncta show variable amount of mGluR7b immunosignal and only few mGluR7b puncta are immunonegative (red line) for Munc13-2 (n

= 152 puncta in 1 rat). (**F-G**) Munc13-2 immunolabeling does not co-localize either with vesicular inhibitory amino acid transporter (VIAAT) (F) or with vesicular glutamate transporter-2 (vGluT2) (G) in rat. Maximum intensity projections of 10 confocal images separated by 1 μm.

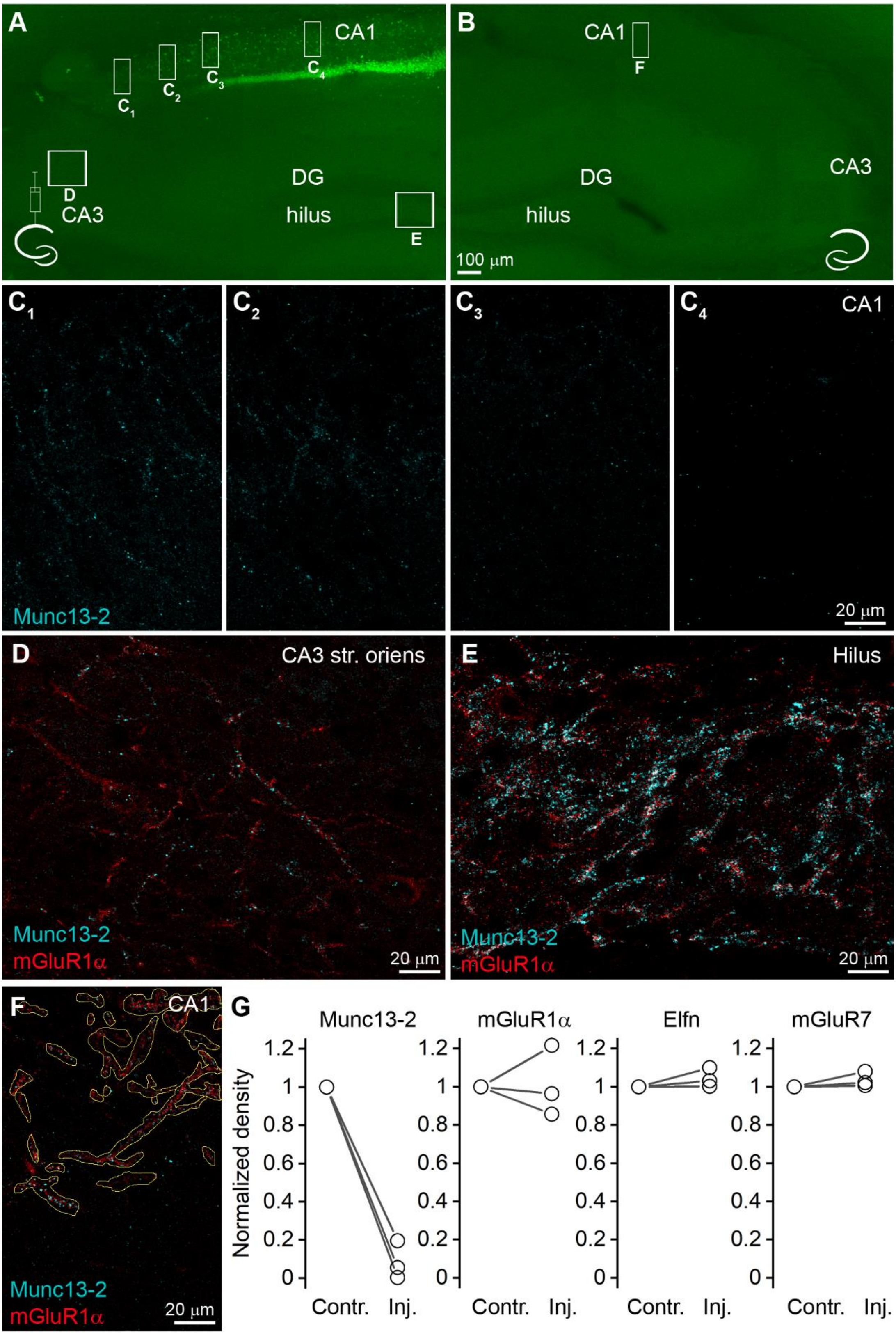

Supplementary Figure 2.

Conditional knock-out of Munc13-2 changes the expression of Munc13-2 only around the injection zone. (**A**) Immunolabeling for Cre shows the restricted virus (AAV8-pAAV Ef1a-mCherry-IRES-Cre)

expression in the middle of the CA1 area. Note the gradual decrease of the signal at the distal part of the CA1 and the total lack in the CA3 area, dentate gyrus (DG) and hilus. Boxes are enlarged in panels C_{1-4} and D, E (**B**) Low magnification image of the contralateral non-injected hemisphere, showing the lack of Cre immunolabeling. Box area is enlarged in panel F. (**C**) Munc13-2 immunolabeling in the CA1 area. Positions of the 4 images are shown in A. Note the gradual increase of the immunosignal outside the injection zone. (**D**) Munc13-2 immunolabeling also shows co-localisation with mGluR1α in the stratum oriens of the CA3 area in the injected hemisphere. (**E**) Prominent Munc13-2 immunolabelling in the hilus around mGluR1α immunopositive dendrites in the injected hemisphere. (**F**) Munc13-2 and mGluR1α double immunolabeling in the non-injected hemisphere. Yellow outline indicates ROIs around mGluR1α positive dendrites where Munc13-2 immunolabeling was quantified. (**G**) Normalized density of Munc13-2, mGluR1α, Elfn, mGluR7 immunolabeling in the middle of the injection zone (Inj.) and in the contralateral hemisphere (Contr., the signal was normalized to the that found in the contralateral hemisphere, n = 3 mice).
